# Application of the Semi-Supervised Learning Approach for Pavement Defect Detection

**DOI:** 10.3390/s24186130

**Published:** 2024-09-23

**Authors:** Peng Cui, Nurjihan Ala Bidzikrillah, Jiancong Xu, Yazhou Qin

**Affiliations:** 1 School of Transportation and Civil Engineering, Nantong University, Nantong 226019, China; 2 Jiangsu Water Source Company Ltd. of the Eastern Route of the South-to-North Water Diversion Project, Nanjing 210018, China; 3 College of Civil Engineering, Tongji University, Shanghai 200092, China; xjc1008@tongji.edu.cn

**Keywords:** semi-supervised, ResNet-18, feature embedding vectors, defect score, explainability, heatmap

## Abstract

Road surface quality is essential for driver comfort and safety, making it crucial to monitor pavement conditions and detect defects in real time. However, the diversity of defects and the complexity of ambient conditions make it challenging to develop an effective and robust classification and detection algorithm. In this study, we adopted a semi-supervised learning approach to train ResNet-18 for image feature retrieval and then classification and detection of pavement defects. The resulting feature embedding vectors from image patches were retrieved, concatenated, and randomly sampled to model a multivariate normal distribution based on the only one-class training pavement image dataset. The calibration pavement image dataset was used to determine the defect score threshold based on the receiver operating characteristic curve, with the Mahalanobis distance employed as a metric to evaluate differences between normal and defect pavement images. Finally, a heatmap derived from the defect score map for the testing dataset was overlaid on the original pavement images to provide insight into the network’s decisions and guide measures to improve its performance. The results demonstrate that the model’s classification accuracy improved from 0.868 to 0.887 using the expanded and augmented pavement image data based on the analysis of heatmaps.

## 1. Introduction

The road network has rapidly expanded over recent decades due to economic and population growth in China. This extensive infrastructure development necessitates ongoing monitoring and maintenance to ensure road longevity and safety. Various factors, including severe weather, vehicle overloading, and natural aging, can degrade road surfaces, leading to driver discomfort and potential safety hazards. To mitigate these issues, real-time road quality monitoring is critical for detecting existing and potential pavement defects, such as cracks, potholes, rutting, and raveling, in a timely manner. However, developing an effective approach for the accurate and robust identification of pavement anomalies poses significant challenges. These challenges stem from the diversity of road distresses and image-related factors, such as road markings, shadows, and variations in illumination.

The recent literature on this topic has aimed to provide cost-effective solutions for efficiently and accurately detecting and locating road surface defects. Based on the techniques and equipment used, these studies can be roughly classified into three categories: three-dimensional (3D) reconstruction methods, vision-based methods, and vehicle response-based methods. In 3D reconstruction methods, 3D pavement image data are typically obtained from laser scanners installed on inspection vehicles. This method is considered relatively more expensive among the three due to the high cost of laser scanner equipment. Soilán et al. [1] reviewed the laser scanning technique and its application in monitoring the health of the road and railway infrastructures. Ma et al. [2] reviewed the mobile laser scanning technique and its implementation for the extraction and detection of road objects. With the rapid development of light detection and ranging (LiDAR) systems, LiDAR scanning techniques are increasingly used to capture precise spatial information of target objects and to establish digital twins of real objects using 3D point clouds. Mattes et al. [3] proposed a semi-supervised generative adversarial network (GAN) for 2D road images rendered from LiDAR point cloud data collected by mapping vehicles. They reported that their method was robust and reliable on two road image datasets. The paper also implements a pipeline for rendering 3D point clouds of roads for future use. Hu and Furukawa [4] presented a method to reconstruct three-dimensional road surfaces for defect detection using overlapping images. On-site tests showed that their proposed method achieved over 94% precision, accuracy, and recall.

With the rapid development of computer vision techniques, more and more research has been conducted on vision-based defect detection [5]. Compared to manual visual inspection, automated visual inspection is more efficient and accurate, as it reduces human error. Based on the manner of data labeling, these approaches can be classified into supervised, unsupervised, and semi-supervised learning. Ren et al. [6] presented a semi-supervised learning approach for detecting pavement distress using a GAN capable of detecting pixel-level anomalies in images, achieving an accuracy of 80.75%. Another method was proposed by [7] for detecting asphalt pavement bleeding using a one-class support vector machine (SVM) algorithm, with a reported F1-score of 97.29%. Han et al. [8] introduced a two-stage segmentation framework for detecting pavement cracks using both supervised and unsupervised strategies, reporting that their model surpassed other state-of-the-art neural networks in intersection over union (IoU) scores. Zhao et al. [9] proposed two approaches for detecting road surface anomalies and estimating vehicle speed based on distributed fiber-optic sensing, confirming the feasibility of their methods. Chacra and Zelek [10] introduced a pattern recognition method using street view images, employing the Fisher vector formulation and SVM to encode and classify image data. Their model was also capable of assessing the severity of road quality. Wang et al. proposed a pavement defect segmentation method using a relevance-aware and cross-reasoning approach [11], while Lin et al. [12] introduced a pavement anomaly detection method based on transformers and self-supervised learning. Kaddah et al. [13] presented a method to address two types of pavement anomalies: road marking degradation and pavement cracking. Their approach utilized color segmentation, inverse perspective mapping, optical correlation, and geometric recognition techniques. More literature on this topic can be found in [14]. Bello-Salau et al. [15] reviewed vision-based approaches for road anomaly detection, aiming to develop a robust processing approach to tackle this challenge, as did [16]. Overall, vision-based approaches for pavement defect detection are widely used and have succeeded in many areas. However, it is important to note that these approaches are often susceptible to background clutter, such as shadows or illumination variations, which can limit their robustness. Additionally, vision-based approaches typically require large amounts of image data for training deep learning models, which can be difficult or even impossible to collect in some fields. Labeling training data is also laborious and time-consuming. Moreover, how to explain the detection results from neural networks is rarely addressed in existing studies, yet it is crucial for understanding the underlying mechanisms of the networks and improving their performance in the future.

The vehicle response-based approach involves measuring the dynamic response of a vehicle using sensors such as smartphones, accelerometers, gyroscopes, and more, and the wavelet transformation is usually utilized to transform the one-dimensional signals into two-dimensional ones [17,18,19]. Sattar et al. [20] proposed an unsupervised approach for real-time detection of road surface anomalies using linear accelerometer data collected from smartphone apps and the K-means clustering algorithm. They explored three cases, achieving reasonable accuracy with their model. Similar research has been conducted using acceleration or gyroscope signals collected from smartphones, with clustering algorithms used to classify normal and anomalous road conditions [21,22,23,24,25,26,27,28]. Liu et al. [29] introduced a response-based method to detect road anomalies via an automated vehicle, focusing on the relationship between driving comfort, vehicle speed, and road anomalies (specifically height differences in the pavement). The results showed that the root mean square (RMS) error of the anomaly evaluation was approximately 0.63 cm. An unsupervised, tiny machine learning approach for detecting road anomalies was proposed by [30], who reported that the F1-score of the classifier model reached 0.78 for the second driver. Martinez-Ríos et al. [31] used continuous wavelet transform (CWT) to convert monitored vehicle response signals and then utilized a convolutional neural network (CNN) to identify transverse pavement cracks using a transfer learning approach. Similar CWT-based research was presented by Xie et al. [32]. Wang et al. [33] proposed a framework using accelerometers and gyroscopes attached to vehicles to detect road surface defects, achieving real-time monitoring of road anomalies in Taipei city via the Internet of Things. Dib et al. [34] reviewed techniques for detecting negative road anomalies (e.g., potholes and cracks), highlighting their strengths and weaknesses. Martinez-Ríos et al. [35] reviewed vibration-based methods for detecting road surface anomalies, focusing on preprocessing steps, threshold setting, feature extraction techniques, and deep learning methods. Additional related research can be found in [36].

Upon reviewing the existing literature, the vehicle response-based method is identified as the most cost-effective because sensors like gyroscopes and accelerometers can easily be embedded in smartphones. However, the relationship between a vehicle’s response and pavement distress is complex and influenced by several factors, such as vehicle type, smartphone type, vehicle speed, and sensor placement. These limitations result in discrete outcomes that may not accurately reflect the actual pavement condition.

Semi-supervised learning aims to reduce labeling costs while enhancing a model’s performance and generalization. It involves training with a relatively small amount of labeled data alongside a large amount of unlabeled data. The labeled data guide the model’s training in the desired direction, while the unlabeled data help the model explore and learn the underlying structure and patterns within the data. Given the abundance of easily obtainable unlabeled data in the real world, semi-supervised learning offers significant advantages over other approaches.

**Figure 1:**
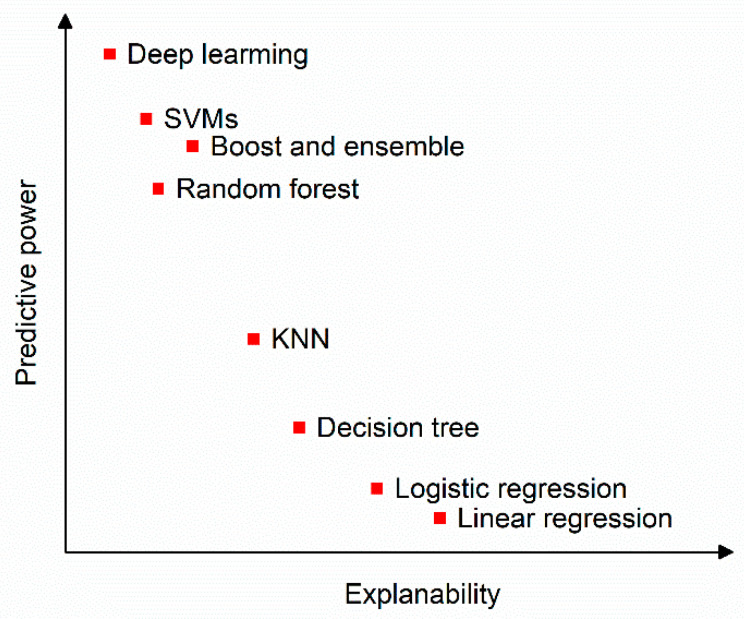
Diagram of the explainability of the intelligent models and their predictive power (courtesy of online).

Another important issue to consider in this paper is the explainability of the neural network model’s decisions for test images. Explainability is crucial for gaining insights into specific issues when using artificial intelligence techniques, especially with deep CNNs. However, to the best of the authors’ knowledge, relatively few works have addressed this aspect. Neural networks, particularly deep neural networks, often function as “black boxes”, providing exact detection or accurate classification results but without offering explanations for those results. Figure 1 illustrates the power of various intelligent models, including deep learning and machine learning, etc., in relation to their explainability.

Figure 1 clearly shows that deep learning models have the most power but the least explainability, while linear regression models are the simplest and easiest to understand but have the lowest power. Therefore, providing a reasonable explanation of the results from deep learning models is crucial for understanding the underlying logic of the neural network and potentially improving its performance in the future.

**Figure 2:**
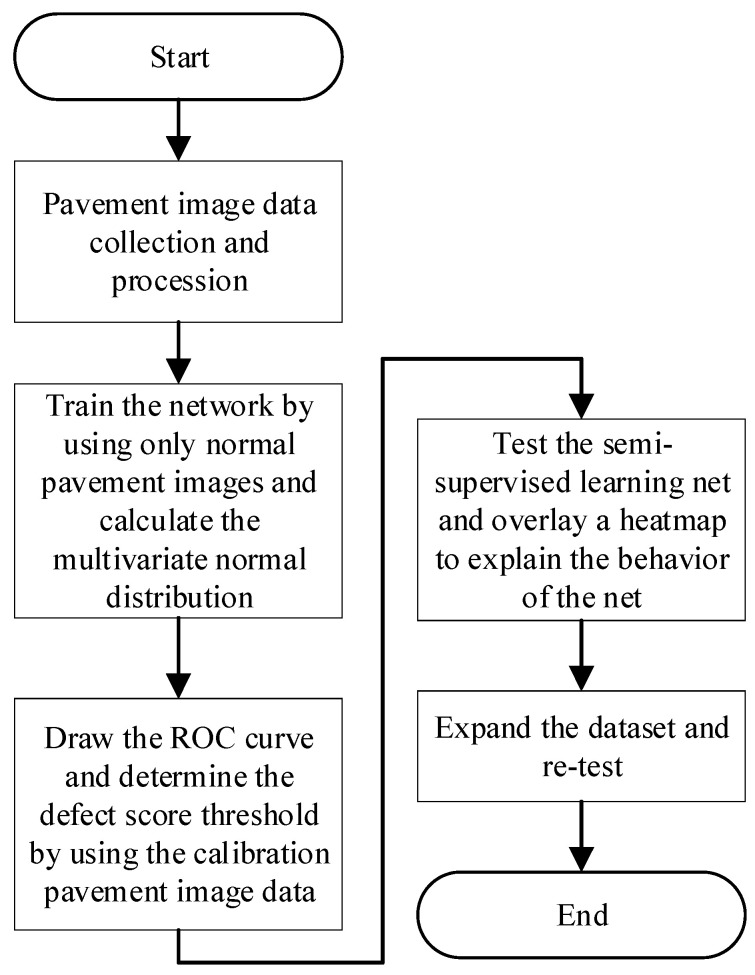
The flowchart of the study.

Based on the preceding discussion, this study’s innovations can be summarized in two key points. First, we employ a semi-supervised approach using a one-class training strategy to reduce the effort required for collecting and labeling anomalous pavement image data. Second, we overlay heatmaps generated from defect score maps onto the original pavement images, providing visual explanations for the decisions made by the trained neural network. The primary objective of this study is to apply this one-class semi-supervised learning method for pavement defect detection while enhancing result interpretability through heatmap visualization. The methodological approach of this study is illustrated in Figure 2.

The remainder of the paper is structured as follows: the relevant theory is introduced in Section 2, data augmentation and model application are described in Section 3, followed by the results and discussion in Section 4. Finally, the conclusion is summarized in Section 5.

## 2. Methodology

### 2.1. Briefing of ResNet-18

ResNet-18 is part of the residual network family, designed to address the degradation problem in deep CNNs. It was first proposed by He et al. [37] and features innovative identity shortcut connections that bypass one or more layers, allowing the network to learn residual functions rather than directly fitting the underlying mapping. The schematic diagram of the building block in ResNet-18 is shown below.

**Figure 3:**
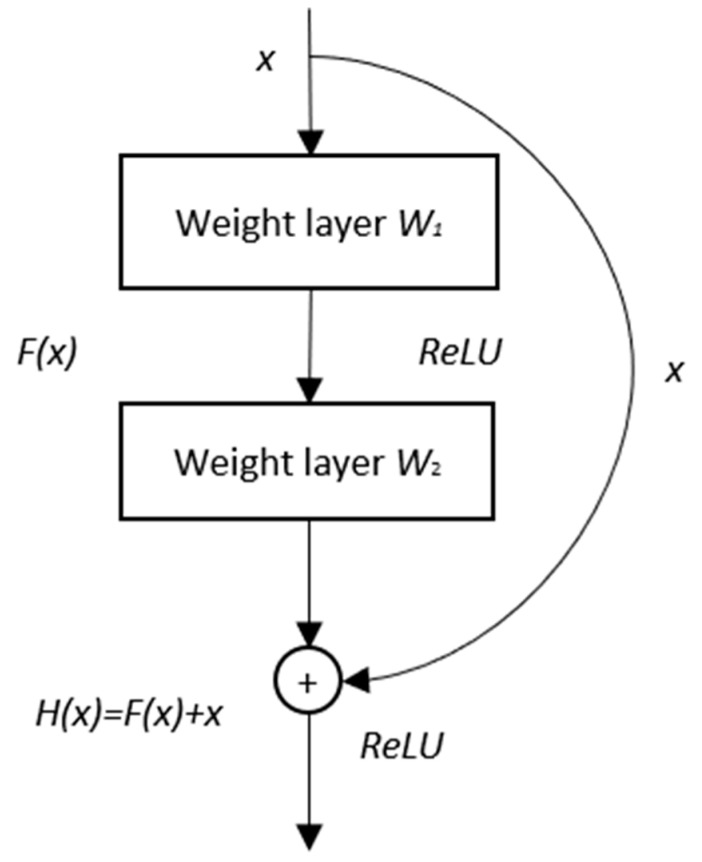
Schematic diagram of the building block of ResNet-18.

In Figure 3, *x* denotes the input to the first weight layer of the building block in the ResNet-18, while *H*(*x*) is the desired underlying mapping, and *F*(*x*) represents the residual function in relation to *H*(*x*) and *x*. The curved arrow in Figure 3 is the identity shortcut connection, which transforms the plain network into a residual network, simplifying the learning process and accelerating convergence [37,38,39].

**Figure 4:**
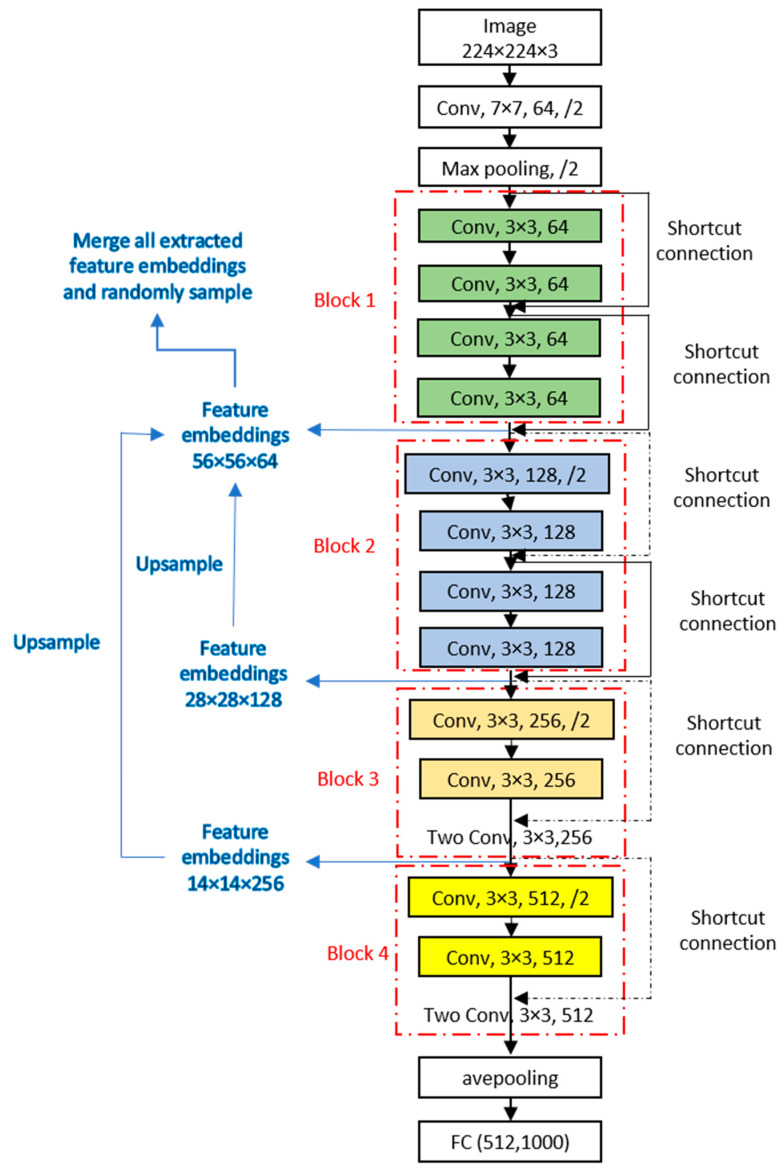
The architecture of the ResNet-18 network and the retrieval of feature embeddings (dash-dot lines denote change dimension; two convolutional layers are omitted in blocks 3 and 4 due to space limitation. Conv in Figure 4 stands for convolutional layer, 3×3 is the size of filters, 64 is the number of filters, and /2 denotes a stride of 2).

ResNet-18 consists of 18 layers used to learn input features. The first convolutional layer contains 64 filters, each of size 7 by 7, with a stride of 2, followed by a max pooling layer. The network then includes four blocks, each with two units of paired convolutional layers. The architecture concludes with an average pooling layer and a fully connected layer. The ResNet-18 architecture is illustrated in Figure 4, with the core steps for retrieving patch feature embeddings using the patch-distribution-based method shown on the left side of the diagram.

All in all, the identity shortcut connections in ResNet-18 streamline the deep CNN network, balancing high performance with time and space complexity. Recently, ResNet-18 has been widely used in image classification [40], image feature extraction [41], object detection, and segmentation [42].

### 2.2. Application of the Patch-Distribution-Based Method

The patch-distribution-based method proposed by Defard et al. [41] is highlighted on the left side of Figure 4. This method was validated on the Machine Vision Technologies Anomaly Detection (MVTec AD) and ShanghaiTech Campus (STC) datasets, where it was reported to outperform other state-of-the-art methods in terms of accuracy and simplicity. The main advantages of this method are twofold. First, it uses a pretrained ResNet-18 to extract spatial patches from the input images, eliminating the need for retraining on application-specific data. This makes the method time-efficient and easy to apply to new fields. Second, as a one-class learning method, it requires only normal image data to learn and extract patterns from the training data’s structure. Given these advantages, we employ this method to distinguish defect pavement images from normal ones.

As shown in Figure 4, blocks 1, 2, and 3 of ResNet-18 are used to retrieve spatial patches. Block 1 outputs 64 patches of size 56 by 56, block 2 provides 128 patches of size 28 by 28, and block 3 yields 256 patches of size 14 by 14. To ensure consistent dimensions across different scales, an upsampling approach is applied to align the patches. Through the upsampling operation, all retrieved patches are resized to 56 by 56. The retrieved matrix is then merged along the channel dimension, forming a total of 448 channels per image. The 56×56 matrix is subsequently reshaped into a vector with 3136 entries, forming the spatial feature embeddings. This process of upsampling and merging enables the extraction of multi-scale resolutions, capturing both fine-grained details and global context in the patch embeddings.

The model is trained using only collected normal pavement images, and the distribution pattern of the patch embedding vectors from the training set images is learned. It is worth noting that we randomly selected only 110 channels for pattern recognition from the total of 448 channels in the extracted patch feature embeddings to accelerate convergence while maintaining nearly the same classification accuracy. Here, we assume that the multivariate normal distribution *N*(*µ_ij_*, ∑*_ii_*) of the patch embeddings of the training dataset exists at position (*i*, *j*), and if the embedding vector is denoted as *X_ij_*, then the mean and covariance of the patch embedding vectors at position (*i*, *j*) across all training images can be expressed as:

(1) $$\mu_{ij}=\frac{1}{N}\sum_{k=1}^{N}X_{ij}^{k}$$

(2) $$\sum_{ij}=\frac{1}{N-1}\sum_{k=1}^{N}(X_{ij}^{k}-\mu_{ij}){\left(X_{ij}^{k}-\mu_{ij}\right)}^{T}+\varepsilon I$$

Here, *µ_ij_* and ∑*_ij_* denote the mean and covariance array of the patch embedding vectors at position (*i*, *j*) across all training images, respectively. *I* represents the identity matrix, *N* is the total number of training images, and *k* denotes the *k*th image, where *k* ∈ [1, N]. The term *εI* is a regularization term added to ensure the covariance matrix is full rank and invertible, with the regularization constant set to *ε* = 0.02.

The Mahalanobis distance (MD) is used to evaluate the difference between each patch embedding vector of the pavement images in the test set and the corresponding learned normal distribution from the training set. Unlike the Euclidean distance, the MD accounts for the covariances between embedding vectors. Therefore, if a pavement image contains defects such as potholes, cracks, rutting, or bumps, the MD for that image will be larger compared to that of a normal pavement image. The MD is calculated as follows:

(3) $$M(X_{ij})=\sqrt{{\left(X_{ij}-\mu_{ij}\right)}^{T}\sum_{ij}^{-1}(X_{ij}-\mu_{ij})}$$

where *X_ij_* is the patch embedding vector extracted from the test set images. The high MD means a high probability of defects on the pavement image. Then, the matrix of MD is visualized into a defect score map in order to localize the anomalies in the pavement image. Next, the averaged defect score for each image is calculated for the classification of the pavement images.

The calibration procedure aims to determine the defect score threshold that separates normal pavement images from defective ones. The receiver operating characteristic (ROC) curve is utilized, and the area under the curve (AUC) value is calculated. The larger AUC value indicates better performance of the model. The optimal defect score threshold is selected based on the maximum Youden index metric using the calibration dataset. This threshold maximizes the true positive rate while minimizing the false positive rate. Once the defect score threshold is established, test set images can be classified accordingly.

One downside of deep CNNs is that their results are often difficult to interpret. As shown in Figure 1, while deep CNN models achieve high accuracy, they are also the least explainable. In this study, a heatmap is overlaid on the original pavement image to provide insight into how the neural network makes decisions to differentiate between defect and normal pavement images during the testing phase and to highlight what the trained neural network focuses on. Exploring the explainability of the deep CNN model can be beneficial for improving the model’s performance in future research.

## 3. Data Augmentation and Model Application

### 3.1. Pavement Image Data Collection and Augmentation

**Figure 5:**
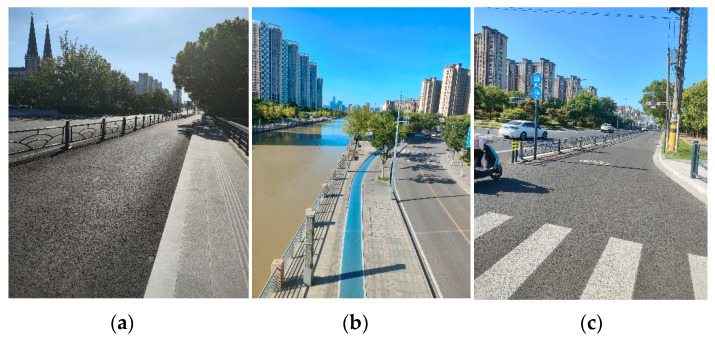
Pavement image data collected from various roads: (**a**) Qingnian Road; (**b**) Riverside Road; and (**c**) Tongjing Dadao.

Pavement image data were collected in Chongchuan District, Nantong, Jiangsu, China, including areas along Qingnian Road, Riverside Road, and Tongjing Dadao, as shown in Figure 5. These locations were chosen because Nantong Metro Line 2 runs directly beneath Qingnian Road in the same longitudinal direction. Monitoring pavement anomalies on these road surfaces above Metro Line 2 is particularly significant for evaluating the dynamic effects of the subway on nearby infrastructure. The dataset includes 1000 labeled positive images with defects and 2000 labeled negative images without defects.

**Figure 6:**
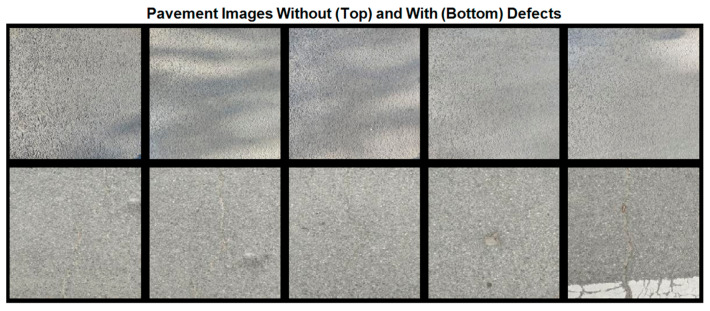
Some samples of the pavement images (top: images without defects; bottom: images with defects).

Figure 6 displays some samples of the pavement images. The five images in the first row show pavement without defects, while the five images in the second row depict various types of defects. Due to the different times at which these photos were taken, they exhibit varying illumination conditions and some shadows in the normal images. These factors can inevitably affect the classification accuracy of the neural network and will be discussed in the subsequent section. Notably, the middle image in the second row features cracks that are extremely narrow, making them difficult to recognize even with the naked eye. This characteristic increases the likelihood of misclassification by the neural network.

The collected pavement image data are divided into three parts for training, calibration, and testing. Specifically, 300 normal pavement images (negative class only) are used for training. For calibration, 100 pavement images without defects and 100 pavement images with defects are selected. For testing, 300 pavement images without defects and 300 pavement images with defects are used. In summary, there are 300 normal pavement images for training, 200 mixed pavement images (100 images with defects and 100 images without) for calibration, and 600 mixed pavement images (300 images with defects and 300 images without) for testing. Prior to training, calibration, and testing, all randomly selected pavement images are preprocessed by resizing and center-cropping to a size of 224 by 224 pixels.

### 3.2. Model Application

As stated in Section 2.2, spatial patches of the pavement images are retrieved from three layers of ResNet-18. To standardize the spatial resolution of feature patches from different layers, upsampling is used to resize all extracted patches to 56 by 56 pixels. These extracted feature patches are then aggregated and reshaped into feature embedding vectors. To avoid information redundancy and reduce computational burden, a random selection process is conducted, retaining about a quarter of the total feature embedding vectors. With the selected feature embedding vectors for the training data, the mean *µ_ij_* and covariance ∑*_ij_* of the normal distribution are calculated based on the training data. The calibration pavement dataset, comprising 100 normal and 100 defect images, is used to set a defect score threshold. The Mahalanobis distance (MD) is chosen as the metric for measuring differences between the target images and normal images due to its extra consideration of covariance, which yields more accurate results compared to the Euclidean distance. The defect score map for each pavement image is generated, and the mean defect score for the images is calculated subsequently. The distribution of these scores for the calibration dataset is displayed in Section 4.

The receiver operating characteristic (ROC) curve is used to determine the defect score threshold. The area under the curve (AUC) is computed, and the defect score threshold is selected based on the maximum Youden index. This threshold corresponds to the maximum distance between the ROC curve and the random chance line, which represents the occurrence chance of 50%. The test dataset includes 300 normal and 300 defect pavement images randomly selected from the overall pavement image dataset. Following the same procedure used for the calibration dataset, the MD between the embedding vectors of the test images and the normal distribution of the training images is calculated, and the defect score map for each test image is generated. The defect score map is then transformed into a heatmap later to visualize the scores in the images. This heatmap is overlaid on the original pavement images to explain the ResNet-18 model’s decisions and what its focus is to identify potential areas for improving the network’s performance in future work.

## 4. Results and Discussion

### 4.1. Results

**Figure 7:**
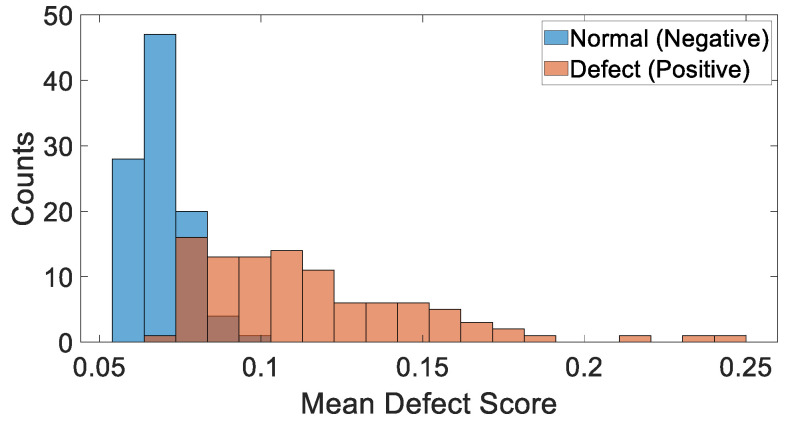
Histogram of the mean defect score for the calibration pavement dataset.

Figure 7 shows the histogram of the mean defect score per pavement image for the calibration dataset. The defect score is derived from the MD between the normal distribution of the training pavement data and the feature embedding vectors of the calibration dataset. Figure 7 illustrates that ResNet-18 effectively differentiates between defect and normal pavement images. Specifically, the mean defect scores for normal pavement images are generally below 0.08, while defect images typically have higher scores, mostly exceeding 0.08. However, there is some overlap between the two classes, with a small number of samples in the two groups falling within the range of 0.06 to 0.10 along the mean defect score axis.

**Figure 8:**
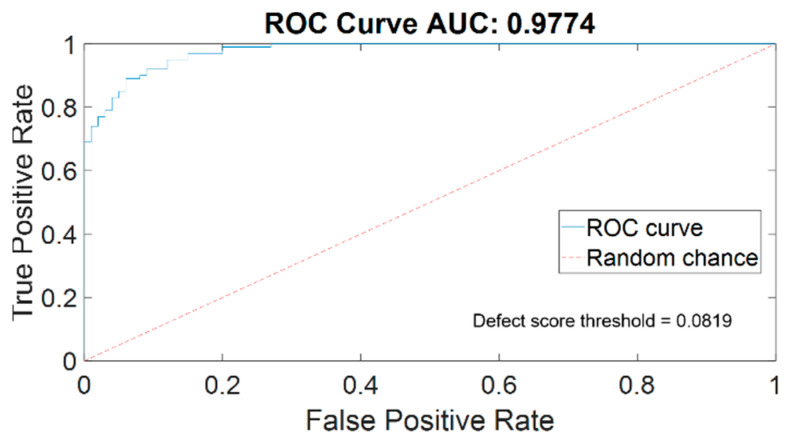
The ROC curve for the calibration pavement dataset.

The ROC curve for the neural network is shown in Figure 8. The AUC value is 0.977, which indicates a relatively strong performance of the classification neural network. As mentioned earlier, the maximum Youden index is used to determine the optimal defect score threshold of 0.0819 for distinguishing between defect pavement images and normal ones, as depicted in Figure 8. The red dashed line in the figure represents the random chance level with a probability of 50%.

**Figure 9:**
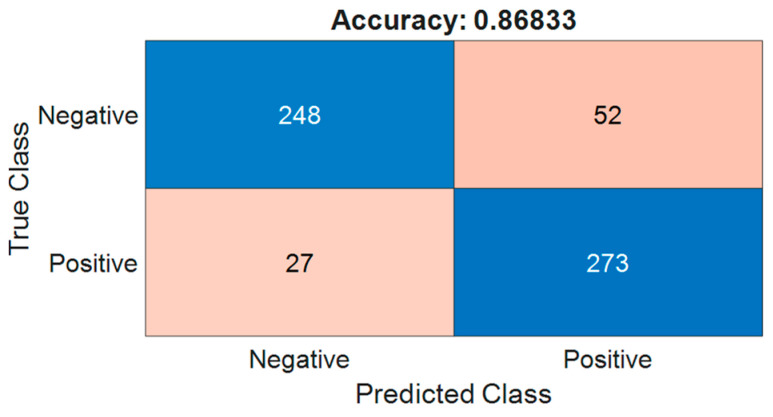
The confusion matrix for the testing pavement dataset.

After determining the defect score threshold, we apply it to the test dataset to differentiate between anomalous and normal pavement images. The confusion matrix for the testing pavement set is shown in Figure 9. From the figure, the classification accuracy of the ResNet-18 on 600 test pavement images is 0.868. There are a total of 79 misclassified images, including 52 false positives and 27 false negatives. The reasons for these misclassifications will be analyzed using the heatmap in the following sections. Overall, the classification accuracy of this semi-supervised model is considered acceptable.

Another important topic that will be discussed in this paper is understanding how ResNet-18 makes decisions to distinguish between defective and normal pavement images. This aspect is often overlooked in the existing literature but is crucial for potentially improving the model’s performance if we understand how it operates and what it focuses on in the images. We will present the classification results by using ResNet-18 along with heatmaps overlaid on the corresponding original pavement images. These heatmaps are generated based on calculated defect scores based on MD values, allowing us to directly highlight potential pavement defects in the images. The color intensity in the heatmaps corresponds to areas where the model detects higher probabilities of defects, providing insight into the CNN’s decision-making process. For consistency, the heatmap display range is defined the same for all testing set images: the minimum value is set to 0, and the maximum value is set to the 90th percentile of the maximum defect scores from all calibration data.

**Figure 10:**
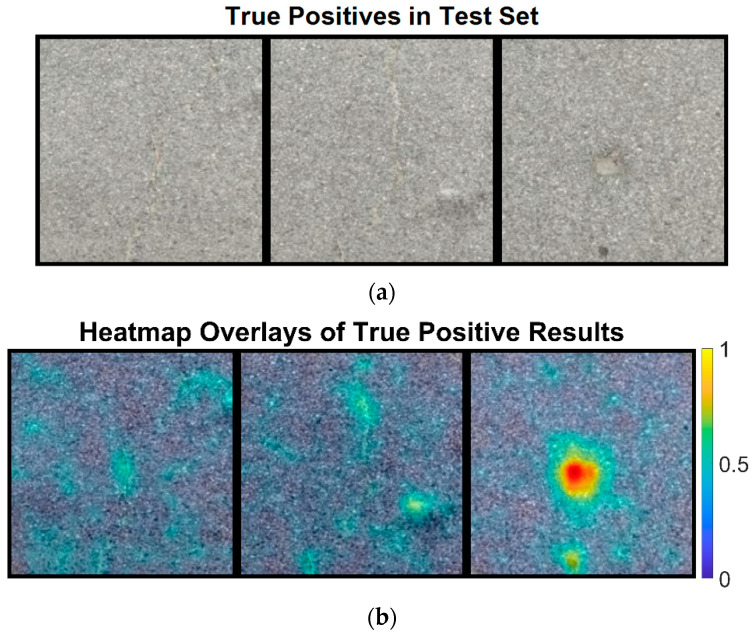
The original image and heatmap image for the true positive pavement images: (**a**) the original pavement image and (**b**) the heatmap image of the true positive results.

First, we will explain the true positive results classified by ResNet-18 using the heatmaps. These results represent correctly classified pavement images with defects. The heatmaps are based on the defect score map, and true positive results refer to pavement images predicted to have defects that indeed have defects, as shown in Figure 10a.

Figure 10b displays heatmaps overlaid on the corresponding original pavement images that were correctly classified as true positive results. It is evident from Figure 10b that with semi-supervised learning, the ResNet-18 network effectively captures the exact locations of the defects, as indicated by the hot colors. This demonstrates that the ResNet-18 not only classifies pavement images as normal or defective but also locates the defects accurately. The mean defect scores for these three images are 0.0826, 0.0825, and 0.0894, all exceeding the threshold of 0.0819.

**Figure 11:**
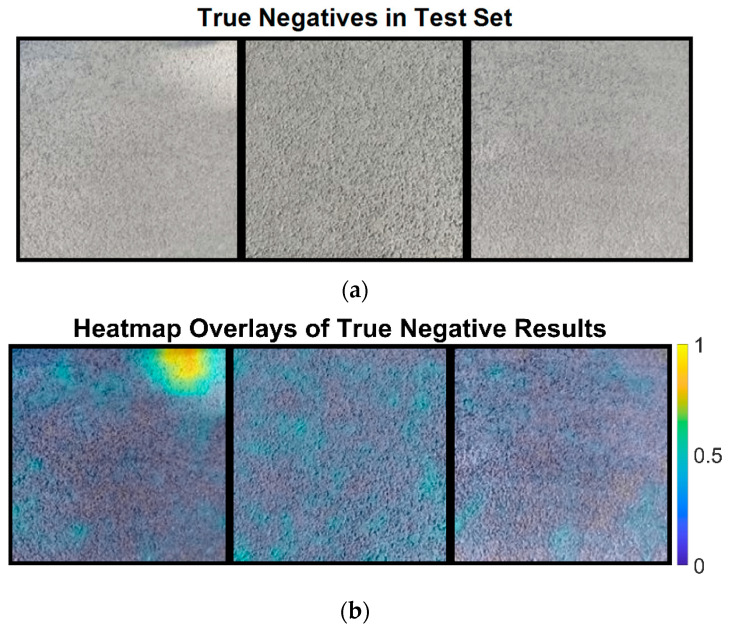
The original image and heatmap image for the true negative results: (**a**) the original pavement images and (**b**) the heatmap image of the true negative results.

Next, we analyze the true negative results classified by the semi-supervised learning ResNet-18, as shown in Figure 11a. True negative results indicate that the network has correctly identified pavement images without defects. Figure 11b presents heatmaps overlaid on these correctly classified images.

Figure 11b shows that most of the heatmap colors are cool, indicating no defects in the pavement images. However, the first image in Figure 11b exhibits hot colors in the top right corner due to illumination variation. This suggests that changing illumination in local regions might affect the neural network’s decision, even though the pavement image is correctly classified. The mean defect scores for these images are 0.0766, 0.0787, and 0.064, all below the threshold of 0.0819.

**Figure 12:**
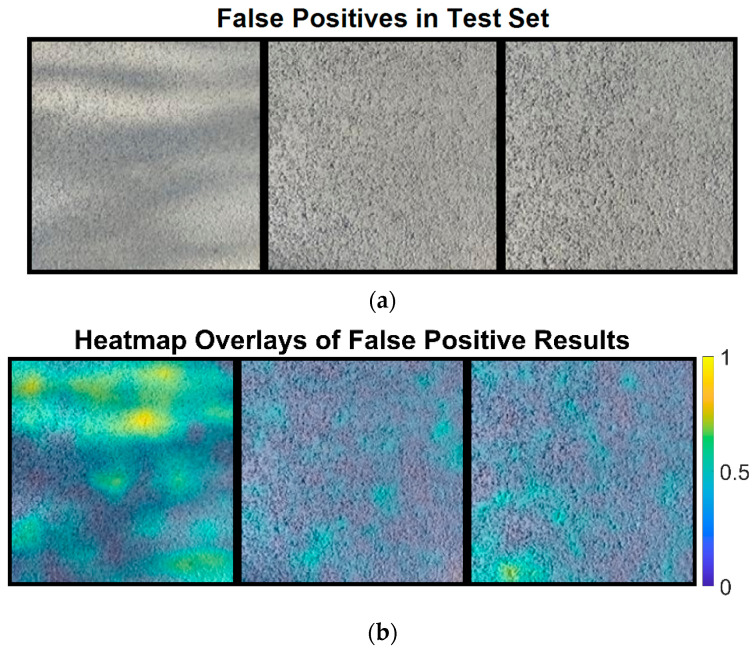
The original images and heatmaps for the false positive results: (**a**) the original images and (**b**) heatmaps for the false positive results.

Then, we analyze the false positive results obtained by using ResNet-18, as shown in Figure 12a. False positive results refer to pavement images without defects that are incorrectly classified as defective ones by the semi-supervised learning network, ResNet-18. Investigating these misclassified images will help identify potential issues in detection and could lead to improvements in the neural network’s performance.

It is evident from Figure 12b that the first pavement image is misclassified primarily due to illumination variations and shadows. The mean defect score values for these images are 0.1123, 0.082, and 0.0889, all exceeding the defect score threshold of 0.0819. The first image, in particular, shows more hot colors and has the highest mean defect score, suggesting that collecting normal pavement images with minimal shadows and illumination variations could help reduce such misclassifications.

**Figure 13:**
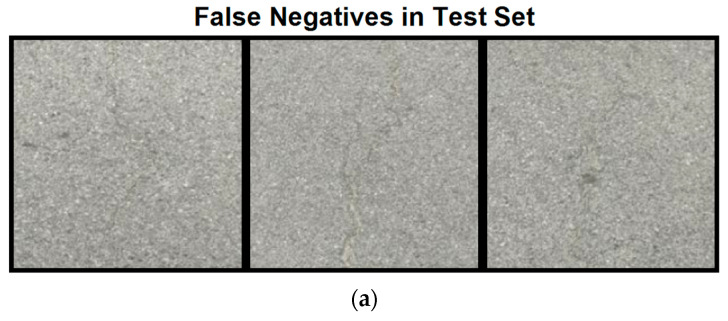

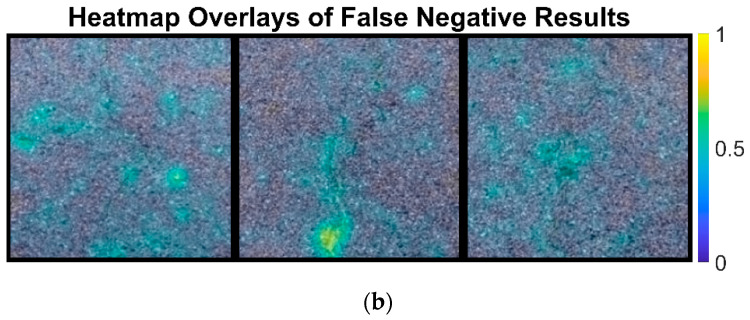
The original images and heatmaps for the false negative results: (**a**) the original pavement images and (**b**) the heatmaps for the false negative results.

Finally, Figure 13 presents the false negative results from the semi-supervised learning neural network model. Figure 13a shows the original pavement images, while Figure 13b displays the corresponding heatmaps overlaid. False negative results occur when pavement images with defects are incorrectly classified as normal ones. In Figure 13a, all three images display cracks, although some are too thin to be detected manually. Notably, in the middle image of Figure 13b, the trained ResNet-18 network detected the crack’s location with hot colors in the heatmap despite the misclassification. This misclassification can be attributed to the selected metric—the mean defect score—which averages the defect scores across the whole image, potentially underestimating defects in localized regions. The mean defect score values for these images are 0.0811, 0.0728, and 0.0775, all below the threshold of 0.0819.

### 4.2. Discussion

Based on the results obtained in the previous subsection, it can be inferred that illumination variations, shadows, and other factors, such as leaves and road markings (e.g., zebra crossings), are major contributors to false positive results. Therefore, it should be taken with caution to collect pavement image data with these factors minimized. Regarding false negative results, we should focus on acquiring pavement images with larger and more noticeable defects to improve the semi-supervised learning neural network model’s performance. Additionally, optimizing the neural network, such as replacing ResNet-18 with a deeper neural network like ResNet-50 or another one, could be another viable approach to enhancing its performance.

**Figure 14:**
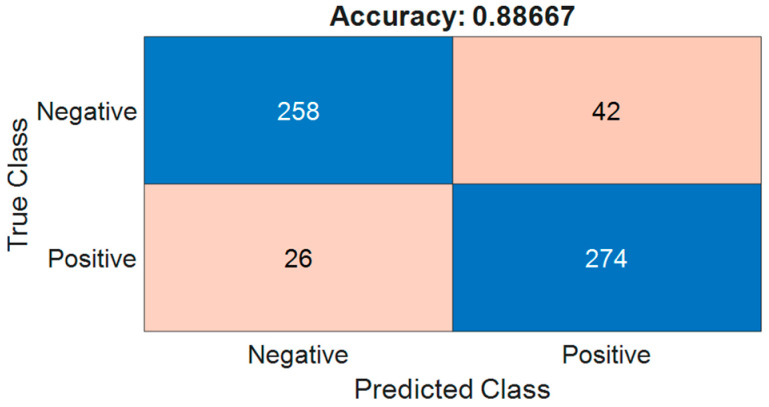
The confusion matrix with the expanded image dataset.

In this study, we have implemented the first approach to enhance the classification accuracy of the semi-supervised learning ResNet-18 model by expanding the pavement image dataset. We collected normal pavement images to supplement the training dataset while avoiding, as much as possible, shadows, leaves, road markings, and illumination variations. Additionally, we enriched the defect dataset with images of larger defects, such as cracks and potholes. The performance of the same neural network was then re-tested using this expanded dataset. The confusion matrix for the expanded pavement image data is displayed in Figure 14.

It is evident from Figure 14 that the classification accuracy of the semi-supervised learning network improved from 0.868 to 0.887 with the expanded pavement image dataset. This improvement demonstrates how using heatmaps to explain the neural network’s decision process can guide the expansion of the dataset. However, due to the limited number of existing studies in this area, further in-depth research is needed.

## 5. Conclusions

In this study, a semi-supervised learning approach is employed to train the ResNet-18 neural network for classifying normal and defective pavement images. Feature embedding vectors are extracted from three layers of the ResNet-18 network, followed by upsampling and reshaping to concatenate them. A multivariate normal distribution is obtained using only one-class training data from normal pavement images. The Mahalanobis distance is then used as a metric to measure the difference between the target images and the obtained normal distribution of the training data. The defect score threshold is determined using the ROC curve based on the calibration pavement image dataset. Subsequently, defect score maps are used to create heatmaps that are overlaid on the original pavement images to explain the neural network’s behavior. Analysis of the neural network’s decisions during the classification and detection process led to the expansion of the pavement image datasets, which improved the classification accuracy of ResNet-18 from 0.868 to 0.887. This case demonstrates that heatmaps can effectively explain classification results in the pavement defect detection case study, reveal the underlying behavior of the neural network, and help address the black-box problem.

However, it is worth noting that the semi-supervised learning method in this study is also susceptible to lighting variations and road markings, similar to other vision-based neural network models. Additionally, given the limited pavement image dataset used in this study, further research is needed to test the model’s robustness and effectiveness.

## Data Availability

The original contributions presented in the study are included in the article; further inquiries can be directed to the corresponding authors.

## References

[1] Soilán M., Sánchez-Rodríguez A., del Río-Barral P., Perez-Collazo C., Arias P., Riveiro B. (2019). Review of Laser Scanning Technologies and Their Applications for Road and Railway Infrastructure Monitoring. Infrastructures.

[2] Ma L., Li Y., Li J., Wang C., Wang R., Chapman M.A. (2018). Mobile Laser Scanned Point-Clouds for Road Object Detection and Extraction: A Review. Remote Sens..

[3] Mattes P., Richter R., Döllner J. (2023). Detecting road damages in mobile mapping point clouds using competitive reconstruction networks. AGILE GIScience Ser..

[4] Hu Y., Furukawa T. (2020). Degenerate near-planar 3D reconstruction from two overlapped images for road defects detection. Sensors.

[5] Cui P., Qin Y. (2024). Introducing Methods for Analyzing and Detecting Concrete Cracks at the No. 3 Huaiyin Pumping Station in the South-to-North Water Diversion Project in China. Buildings.

[6] Ren R., Shi P., Jia P., Xu X. (2023). A semi-supervised learning approach for pixel-level pavement anomaly detection. IEEE Trans. Intell. Transp. Syst..

[7] Daneshvari M.H., Mojaradi B., Ameri M., Nourmohammadi E. (2024). Automation detection of asphalt pavement bleeding for imbalanced datasets using an anomaly detection approach. Measurement.

[8] Han C., Yang H., Ma T., Wang S., Zhao C., Yang Y. (2024). CrackDiffusion: A two-stage semantic segmentation framework for pavement crack combining unsupervised and supervised processes. Autom. Constr..

[9] Zhao J., Wang H., Chen Y., Huang M.-F. (2022). Detection of road surface anomaly using distributed fiber optic sensing. IEEE Trans. Intell. Transp. Syst..

[10] Abou Chacra D., Zelek J. Municipal infrastructure anomaly and defect detection. Proceedings of the 2018 26th European Signal Processing Conference (EUSIPCO).

[11] Wang Y., Niu M., Song K., Jiang P., Yan Y. (2023). Normal-knowledge-based pavement defect segmentation using relevance-aware and cross-reasoning mechanisms. IEEE Trans. Intell. Transp. Syst..

[12] Lin Z., Wang H., Li S. (2022). Pavement anomaly detection based on transformer and self-supervised learning. Autom. Constr..

[13] Kaddah W., El Bouz M., Ouerhani Y., Alfalou A., Desthieux M. Ongoing Studies for Automatic Road Anomalies Detection on 2D and 3D Pavement Images. Proceedings of the The International Symposium on Optoelectronic Technology and Application (OTA).

[14] Gao Z., Zhao X., Cao M., Li Z., Liu K., Chen B.M. (2023). Synergizing low rank representation and deep learning for automatic pavement crack detection. IEEE Trans. Intell. Transp. Syst..

[15] Bello-Salau H., Onumanyi A., Salawudeen A., Mu’Azu M., Oyinbo A. An examination of different vision based approaches for road anomaly detection. Proceedings of the 2019 2nd International Conference of the IEEE Nigeria Computer Chapter (NigeriaComputConf).

[16] Fan L., Wang D., Wang J., Li Y., Cao Y., Liu Y., Chen X., Wang Y. (2023). Pavement defect detection with deep learning: A comprehensive survey. IEEE Trans. Intell. Veh..

[17] Qin Y. (2023). Investigating bridge vibrational modes under operational conditions using time-frequency analysis. Struct. Infrastruct. Eng..

[18] Qin Y., Fan Y. (2024). Identifying the Bridge Natural Frequency Pattern Under Operational Condition. Int. J. Struct. Stab. Dyn..

[19] Qin Y., Xu J. (2024). Investigation of the modal frequency of a long-span suspension bridge under multiple environmental conditions. Insight-Non-Destr. Test. Cond. Monit..

[20] Sattar S., Li S., Chapman M. (2021). Developing a near real-time road surface anomaly detection approach for road surface monitoring. Measurement.

[21] Seraj F., Zhang K., Turkes O., Meratnia N., Havinga P.J. A smartphone based method to enhance road pavement anomaly detection by analyzing the driver behavior. Proceedings of the Adjunct Proceedings of the 2015 ACM International Joint Conference on Pervasive and Ubiquitous Computing and Proceedings of the 2015 ACM International Symposium on Wearable Computers.

[22] Chuang T.-Y., Perng N.-H., Han J.-Y. (2019). Pavement performance monitoring and anomaly recognition based on crowdsourcing spatiotemporal data. Autom. Constr..

[23] Shtayat A., Moridpour S., Best B., Abuhassan M. (2023). Using supervised machine learning algorithms in pavement degradation monitoring. Int. J. Transp. Sci. Technol..

[24] Xin H., Ye Y., Na X., Hu H., Wang G., Wu C., Hu S. (2023). Sustainable road pothole detection: A crowdsourcing based multi-sensors fusion approach. Sustainability.

[25] Zhan Q., Ding Y., Lei T., Yin X., Wei L., Liu Y., Luo Q. (2024). Abnormal pavement condition detection with vehicle posture data considering speed variations. Sensors.

[26] Martinelli A., Meocci M., Dolfi M., Branzi V., Morosi S., Argenti F., Berzi L., Consumi T. (2022). Road surface anomaly assessment using low-cost accelerometers: A machine learning approach. Sensors.

[27] Chen Y., Zhou M., Zheng Z., Huo M. (2019). Toward practical crowdsourcing-based road anomaly detection with scale-invariant feature. IEEE Access.

[28] Luo D., Lu J., Guo G. (2020). Road anomaly detection through deep learning approaches. IEEE Access.

[29] Liu C., Nie T., Du Y., Cao J., Wu D., Li F. (2022). A response-type road anomaly detection and evaluation method for steady driving of automated vehicles. IEEE Trans. Intell. Transp. Syst..

[30] Andrade P., Silva I., Signoretti G., Silva M., Dias J., Marques L., Costa D.G. An unsupervised tinyml approach applied for pavement anomalies detection under the internet of intelligent vehicles. Proceedings of the 2021 IEEE International Workshop on Metrology for Industry 4.0 & IoT (MetroInd4. 0&IoT).

[31] Martinez-Ríos E.A., Bustamante-Bello R., Navarro-Tuch S.A. (2023). Generalized Morse Wavelets parameter selection and transfer learning for pavement transverse cracking detection. Eng. Appl. Artif. Intell..

[32] Xie J., Niu F., Su W., Huang Y. (2023). Identifying coastal highway pavement anomalies using multiscale wavelet analysis in radar signal interpretation. J. Civ. Struct. Health Monit..

[33] Wang J.-C., Hsieh C.-L., Lee M.-H., Sun C.-H., Wen T.-H., Juang J.-Y., Jiang J.-A. (2024). Research on Monitoring Road Surface Anomalies Using an IoT-Based Automatic Detection System: Case Study in Taiwan. IEEE Trans. Ind. Inform..

[34] Dib J., Sirlantzis K., Howells G. (2020). A review on negative road anomaly detection methods. IEEE Access.

[35] Martinez-Ríos E.A., Bustamante-Bello M.R., Arce-Sáenz L.A. (2022). A review of road surface anomaly detection and classification systems based on vibration-based techniques. Appl. Sci..

[36] Yu Q., Fang Y., Wix R. (2022). Pavement roughness index estimation and anomaly detection using smartphones. Autom. Constr..

[37] He K., Zhang X., Ren S., Sun J. Deep residual learning for image recognition. Proceedings of the IEEE Conference on Computer Vision and Pattern Recognition.

[38] Guo M., Du Y. Classification of thyroid ultrasound standard plane images using ResNet-18 networks. Proceedings of the 2019 IEEE 13th International Conference on Anti-Counterfeiting, Security, and Identification (ASID).

[39] Al-Haija Q.A., Smadi M.A., Zein-Sabatto S. Multi-class weather classification using ResNet-18 CNN for autonomous IoT and CPS applications. Proceedings of the 2020 International Conference on Computational Science and Computational Intelligence (CSCI).

[40] Zhao Y., Zhang X., Feng W., Xu J. (2022). Deep learning classification by ResNet-18 based on the real spectral dataset from multispectral remote sensing images. Remote Sens..

[41] Defard T., Setkov A., Loesch A., Audigier R. Padim: A patch distribution modeling framework for anomaly detection and localization. Proceedings of the International Conference on Pattern Recognition.

[42] Ou X., Yan P., Zhang Y., Tu B., Zhang G., Wu J., Li W. (2019). Moving object detection method via ResNet-18 with encoder–decoder structure in complex scenes. IEEE Access.

